# A developmental arrest? Interruption and identity in adolescent chronic pain

**DOI:** 10.1097/PR9.0000000000000678

**Published:** 2018-09-11

**Authors:** Abbie Jordan, Melanie Noel, Line Caes, Hannah Connell, Jeremy Gauntlett-Gilbert

**Affiliations:** a Department of Psychology, Centre for Pain Research, University of Bath, Bath, United Kingdom; b Department of Psychology, University of Calgary, Alberta Children's Hospital Research Institute, Calgary, Canada; c Department of Psychology, University of Stirling, Stirling, United Kingdom; d Bath Centre for Pain Services, Royal United Hospitals NHS Trust, Bath, United Kingdom; e Faculty for Health and Applied Sciences, University of the West of England, Bristol, United Kingdom

**Keywords:** Adolescence, Chronic pain, Development, Identity, Autonomy

## Abstract

**Introduction:**

Although the pediatric pain literature has explored the role of developmental factors in young children's acute pain, relatively less is known about specific developmental challenges in adolescents with chronic pain.

**Objectives:**

To meet this knowledge gap, this study sought to adopt an idiographic phenomenological approach to examine how adolescents make sense of their own development in the context of living with chronic pain.

**Methods:**

Semistructured interviews were conducted with ten adolescents (12–17 years; 7 females) recruited from a tertiary care pain treatment programme. Interview data were transcribed verbatim and analysed using Interpretative Phenomenological Analysis.

**Results:**

Study findings identified 2 themes: “An externally imposed lens on identity” and “Paradoxes of developmental progress.” The first theme highlighted an understanding of how adolescent identity is perceived. Some adolescents perceived identity as distinct from pain, whereas others perceived identity as part of their chronic pain condition. This theme also detailed how identity was negotiated by adolescents and others through engagement with valued activities. The second theme represented an understanding of how chronic pain disrupts and alters adolescent developmental trajectories at an individual level, suggesting possibilities of enhanced and delayed trajectories. Enhanced trajectories were associated with increased management of emotionally difficult situations and resulted in mastery of complex interpersonal skills.

**Conclusion:**

Findings provided a nuanced understanding of developmental progress in the context of adolescent chronic pain and suggested challenges with drawing normative comparisons. Future research could extend findings by adopting a longitudinal approach to studying adolescent development and eliciting accounts from broader social groups.

## 1. Introduction

Adolescence is a critical developmental period characterised by changes in social, psychological, physical, and cognitive domains, with specific importance ascribed to social transitions from childhood to adulthood.^30^ The developmental literature indicates that the fundamental tasks of adolescence are to achieve autonomy from caregivers, increase dependence on peers, and enhance emotional skill development and regulation.^5,19^ A specific focus has been placed on the study of identity development and how identity formation fits with chronological age and psychosocial maturity.^2^

The pediatric pain field has long argued that children are not simply “little adults” in terms of their perception and experience of pain.^24^ Indeed, a knowledge of development is critical for understanding the experience of pain across infancy, childhood, and adolescence. Adolescence is of particular interest since the onset of pediatric chronic pain spikes during this period.^20^ Although the literature provides a comprehensive understanding of the impact of living with chronic pain on adolescents in numerous domains, little is understood regarding how chronic pain may influence adolescents' perceptions of their development. A minority of qualitative studies have focused on examining how pain impacts specific aspects of development, with studies of peer relationships showing that adolescents with chronic pain report social isolation and difficulties with developing and maintaining peer relationships.^12,13^ With respect to identity formation, examination of narratives of youth with chronic pain revealed that youth report feeling different from peers and perceive that pain acts as a barrier to undertaking future goals.^25^ Other specific work on identity and sense of self in the context of chronic pain has been adult-focused.^27,33^ Such detailed qualitative work has highlighted the complex and typically negative impact of chronic pain on how adults with chronic pain perceive their sense of self and identity, with some individuals demonstrating substantial emotional distress due to a struggle to differentiate between a “good” self and “mean” self.^33^ Although such work has been pivotal in developing knowledge concerning how identity is perceived and understood in chronic pain, this work has been conducted with an adult population and as such, the population typically has a more stable sense of identity even before the onset of chronic pain. Consequently, studying the identity in adolescence is critical, as individuals at this developmental time point are faced with both the challenges of chronic pain to identity but also the normative challenges of identity formation associated with adolescence.

Questionnaire-based research using the developmental subscale of the Bath Adolescent Pain Questionnaire^8^ also identified that adolescents with chronic pain perceive difference between their own developmental progress and that of healthy peers. Findings revealed that the majority of adolescents reported themselves to be less socially developed in particular domains (eg, school progress and independence) than same-aged peers, with the exception of being able to deal with problems.^9^

Although it is acknowledged that the onset of chronic pain is often unanticipated and unexplainable, existing literature demonstrates a dearth of knowledge concerning how the experience of living with chronic pain disrupts and alters adolescents' expectations of their own development. Existing studies have typically adopted a quantitative approach or focused on examining specific facets of development (eg, peer relationships). Consequently, there is no detailed idiographic understanding of the range of challenges that adolescents may face and how they think about these challenges, as they simultaneously negotiate living with chronic pain and undertaking typical adolescent tasks of development. Given that adolescence has been proposed as a sensitive period for development, and pain may be an interruption during this stage, understanding how chronic pain more broadly influences this developmental process is critical.

To date, no study has applied an idiographic, qualitative approach to the broad study of adolescent development in chronic pain. To meet this knowledge gap, the current study applied this approach to explore (1) How individuals with chronic pain negotiate developmental challenges of adolescence; and (2) How chronic pain might interrupt, or advance, developmental milestones during adolescence.

## 2. Methods

### 2.1. Participants

Interviews were conducted with a sample of 10 adolescents (7 females and 3 males) from a specialised UK national pain management treatment centre. Study inclusion criteria included being aged 11 to 18 years and having experienced pain for a minimum duration of 3 months. In this study, participants were between 12 and 17 years of age (mean age = 14.7 years and median age = 15 years) and reported a pain duration between 12 and 137 months (mean pain duration = 51.2 months and median pain duration = 35 months). Adolescent diagnoses included localised idiopathic pain syndrome (n = 2), diffuse idiopathic pain syndrome (n = 3), complex regional pain syndrome type 1 (n = 4), and arthritis (n = 1). See Table 1 for further details concerning the study participants.

**Table 1:**
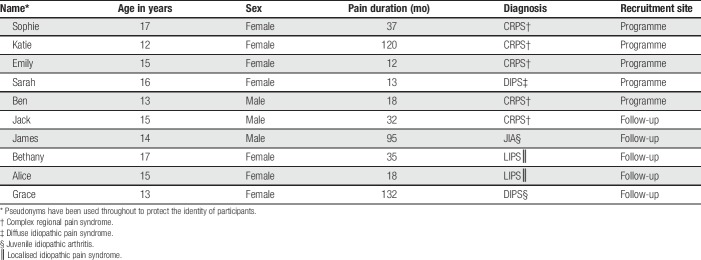
Demographic characteristics of study participants.

In accordance with the approach adopted in Interpretative Phenomenological Analysis (IPA) research, a deliberate effort was made to recruit a small yet homogenous sample of adolescents who had experienced chronic pain for an extended period, were aged 11 to 18 years, and attended treatment at the UK specialist pain management treatment centre. Sample sizes in IPA studies typically range from 1 to 15 participants,^28^ with sample size being guided by the depth of the data and the richness of the data gathered from individual cases. Consequently, we elected to recruit ten adolescents to maximise the ability of the researchers to both compare individual cases and also undertake an in-depth analysis of the data. Fitting with the clear idiographic focus on experience in IPA, no attempt was made to achieve saturation of data, as it is acknowledged that subsequent interviews may produce different data and interpretation compared with previous interviews.^4^

### 2.2. Procedure

After approval from the relevant hospital and university ethics boards was granted, a convenience sample of adolescents was recruited from a 3-week residential in-patient programme and follow-up appointments at the same pain management service. This approach was taken to enable recruitment of adolescents of a sufficient sample of adolescents in a short timeframe.

With regards to recruiting adolescents attending the in-patient programme, the study was introduced by the researcher on the first day of the programme, and adolescents and parents were provided with separate information sheets. Interested adolescents and their parents were jointly asked to complete an opt-in form and return it to the researcher if the adolescent was willing to take part in the study. Recruitment took place across 2 consecutive 3-week programmes, resulting in recruitment of 6 of the ten eligible adolescents attending the 2 selected programmes.

In addition, 6 adolescents attending their 3-month follow-up appointment from a previous in-patient programme were invited to take part in the study. For recruitment through follow-up appointments, letters were first sent to parents/caregivers to explain the nature of the study to parents and included separate information sheets for both parents and adolescents. Parents were asked to share the appropriate information sheet with their child if they were interested in allowing them to consider taking part in the study. If both adolescents and parents were interested in participating in the study, they were asked to jointly return an opt-in form to the researcher to schedule a time for the interview. Of these 6 invited adolescents, 4 adolescents participated in the study.

All participants and their parents/caregiver provided written informed consent before the adolescent being interviewed. Both adolescents and parents/caregivers were informed that the adolescents' participation in the interview was unrelated to any treatment that they would receive. Adolescents also provided verbal consent at the outset of the interview. Interviews took place in a quiet treatment room at the hospital and were conducted by M.I. (n = 6) or A.J. (n = 4). Interviews were digitally recorded, transcribed verbatim, and fully anonymised. At the end of the study, participants were debriefed and given a gift voucher to the value of £5 (United Kingdom) to thank them for their time and participation.

Following good methodological practice when conducting qualitative research, authors' backgrounds in the field of pediatric pain are of relevance and are therefore described briefly below.^10^ At the time of conducting the interviews, M.I. was completing a postgraduate qualification in psychology at the associated university and undertaking a placement in the area of pediatric chronic pain, whereas A.J. had many years' experience of working in the field of pediatric pain. All coauthors are established clinicians or researchers who had worked in the field of pediatric pain for a number of years.

### 2.3. Interview schedule

Semistructured interviews are the exemplary method of data collection in IPA studies,^38^ as they enable the researcher to follow topics of interest as they occur within the interview. Following their suitability for use within IPA research, a semistructured interview schedule was developed to inform the direction and content of the interview discussion. The interview schedule focused on exploring how participants perceived and narrated their experiences of adolescence, including their perceptions of how living with ongoing pain may (or may not) interrupt their ability to engage with the usual tasks of adolescence (eg, identity development). For example, participants were asked “*can you tell me about having chronic pain and your development as a young person?*” and “*what have been the most challenging things about becoming a young person?*” The full interview guide is presented in Table 2.

The interview schedule was developed and discussed between M.I. and A.J. to ensure that time was spent establishing rapport at the start of the interview, and that questions were open-ended and encouraged exploration of selected topics. Prompts were used by the researcher to expand discussion of particular issues where appropriate.^21^ Examples included “*can you tell me a little more about that?*” and prompts for particular areas of discussion if not addressed by the participant freely (eg, school and friends).

**Table 2:**
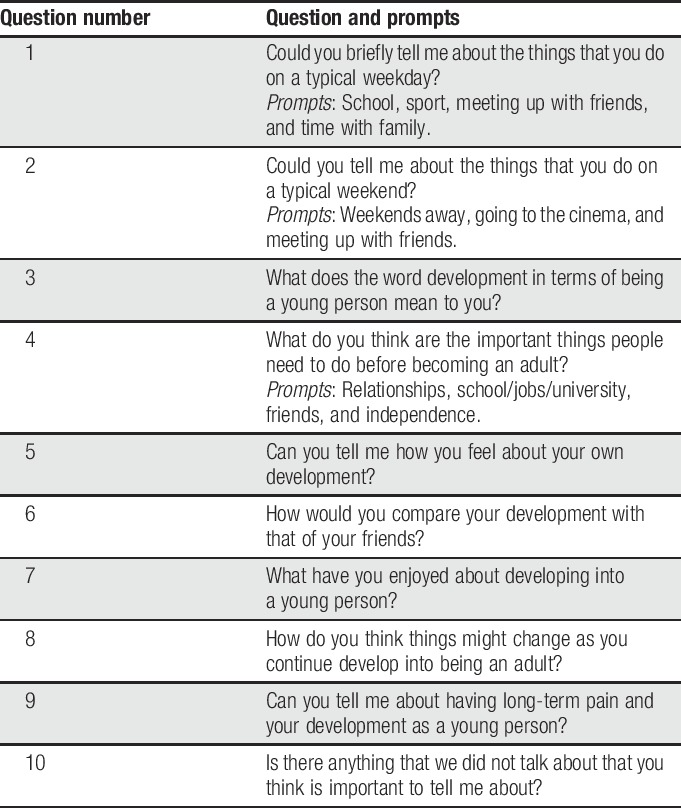
Interview schedule for adolescents with chronic pain.

### 2.4. Data analysis

Interpretative Phenomenological Analysis^32^ was the analytical approach selected for this study. Interpretative Phenomenological Analysis aims to explore the participants' responses in detail, while also recognising the research process as being dynamic, which is in part affected by the interests, conceptions, and concerns of the researcher.^34^ Consequently, IPA does not aim to produce a “true” interpretation of participants' experiences; instead, it recognises that the results of such an analysis are the product of the researchers' engagement with the data.^39^ The process of IPA used in the study followed the detailed description of conducting IPA provided by Smith et al.^34^ Specifically, this entailed addressing each transcript individually through detailed and careful reading of the transcript, identification of initial themes, and exploration of initial themes within that particular transcript. The analytical process was completed through a process of integrating identified themes across all transcripts using QSR International's NVivo software, a computer-assisted qualitative data analysis package. This was achieved by grouping meaningful statements that captured common themes across adolescents' experiences. With regards to transparency and theme development, analyses were originally conducted by A.J. and themes were discussed over multiple meetings with M.N. to establish shared agreement regarding theme content. A mutually agreed on version of analyses was shared and agreed on by all coauthors, providing further credibility checks in terms of analytical interpretation.

### 2.5. Establishing quality in qualitative research

Numerous steps were taken to address the quality of the broader research study and qualitative analyses. Credibility was established through clearly documenting the background and experience of authors in the field of pain, focusing specifically on authors who adopted an interviewing role.^10,31^ In addition, use of QSR International's NVivo software to record memos to document reflections concerning the developing analyses across the duration of the project provided further evidence of credibility. Trustworthiness was established through ensuring that presented quotations were sampled from all participants in the sample, providing discussion of a varied range of participant accounts.^31^ As described in the previous paragraph, authors' involvement in the analytical process was clearly detailed, with all authors confirming that analyses were grounded in the data and included representation of all participants' accounts.

## 3. Results

Analyses highlighted 2 themes that represented the perceptions and experiences of the ten adolescents who participated in the study. Themes were labelled “*an externally imposed lens on identity*” and “*paradoxes of developmental progress*.” Both themes are presented in full below and exemplified by verbatim quotations from participants. Pseudonyms have been used to protect the identity of the participants.

### 3.1. An externally imposed lens on identity

Adolescents in this study discussed how chronic pain was “arresting” their developmental progression. But beyond a sense of interruption of pain on development is a sense of pain “stealing” their identity, the very nature of the individual they are. As described by Sophie below, she feels that others no longer perceive her as an adolescent with individualised preferences and styles but merely as a pain condition.

I think it's difficult when you are in chronic pain to do that because before you can establish your own identity you have been given one and you are already told that you're ill and you have this disease or this condition and that's kind of how you're defined for a while by doctors and then your family and other people is like you will always be—you know—the person with this illness and it's hard to say when you're that person that you have interests in other things and your opinions about things because it's always kind of covered by what you haven't been doing what you have been doing what you've been able to be doing and I think it's difficult to establish an identity when you've already been given one you don't really want (Sophie, 17 years).

This highlights the role of potential iatrogenic effects of clinical diagnosing and encounters in stunting adolescents' identity development (as it pertains to parts of the self that are divorced from pain) in addition to societal and familial influences in constructing adolescent identity in adolescents with chronic pain. In contrast to Sophie's experience, other participants such as Katie did not personally feel defined by chronic pain, nor perceive that others defined them and viewed them in this manner. As Katie describes below, she perceives her identity as being impervious to change as a result of pain.

Even though I have crutches, it doesn't change who I am, like a different personality. I'm not worried because I've got crutches and none of my friends do. When I got this (pain condition) I could see who my real friends are so the ones that call for me, the ones who speak to me (Katie, 12 years).

While expressed positively with the air of pain not impacting on establishing peer relationships, Katie describes appreciating who her “*true friends are.*” This implies that pain may act as a differentiator between true and superficial friends, suggesting that the issue of identity as being influenced by peers is contingent on the closeness of the relationship in question. For some participants, pain acted as a lens through which others viewed them. In such instances, it seemed that the lens itself altered how development was perceived by others, representing it as being both different and stunted. Perceiving and identifying adolescents in the manner of being “stunted” through pain and disability was felt to be restrictive by some adolescents in this study. In the quotation below, Jack purposefully uses the undesirable and socially unacceptable label *“cripple”* to describe how others perceive him. It is informative that this negative term has been specifically selected to represent how Jack feels that he is perceived by others, as it is defined as an offensive term deemed to represent an individual who is unable to use their arms or legs.^29^ Use of this particular term places the focus on Jack's impairment as an entity in its own right and away from the individual (Jack) himself as a person and identity distinct from his pain condition.

It's hard because a lot of people don't understand the situation you are in, so you sort of get stereotyped and pushed to one side as a cripple or somebody like that, and it's hard to sort of pick yourself back up again once you've been called those names, because it's not like you're just living with the pain, it's living with the consequences of the pain and the people—how do people react around you like your friends and teachers and people like that (Jack, 15 years).

But for other adolescents, the interruption of pain on identity development and external perception was more of a personal rather than a social phenomenon. Some adolescents were able to perceive unique aspects of their identity despite experiencing pain, highlighting how their sense of self is not simply defined by pain. Bethany describes, *“I'd say my somewhat sarcastic or a strange sense of humour is kind of a little bit unique to me”* (*Bethany, 17 years*). As Alice describes below, pain has robbed her of many rightful things, yet, both Alice and Bethany hold onto something that is uniquely theirs despite their experience of pain. For Alice, this is a love of dancing: it is something that transcends the pain and is something that she can “hold onto” with regard to enjoyment but also in terms of reflecting a sense of who she is. Alice is more than a pain condition, she is a dancer.

Yeah, because I got a lot of kids in my house and pain takes over our life, dancing is something that's mine and nobody can take it away from me. And when like I've got problems with doctors saying that it's not anything there and I'm not ill. It's the one thing they couldn't take away is dancing, it was like something I had on my own, without my Mum, without anyone, it was something I achieved (Alice, 15 years).

In fact, Alice was adamant that pain should not rob her of her ability to engage with things that defined her and aligned with what she values in life. A sense of quiet determination is evident in Alice's words which follow. *“You are able to be yourself and to be able to do what you want. Even though, like, you've got pain, you still should be able to fill your dreams, your goals, your values and stuff like that,” (Alice, 15 years)*.

Yet, for other adolescent, a sense of being able to hold onto and retain something that was uniquely theirs and that defined them was more difficult with the onset of pain, it slowly slipped out of their grasp over time. In particular, James describes how he is now sadly unable to engage in drumming due to the physical demands of living with his particular pain condition. *“Um, I did play the drums but since this happened [onset of chronic pain], I haven't been able to play the drums, well, I can't do the bass drum either or the high hat pedal” (James, 14 years).*

Consequently, disparities in adolescent experience highlight the individualised nature of complex relationships between pain and identity for adolescents who experience chronic pain. This theme also revealed how this identification may evolve or change with development and the progression of illness.

### 3.2. Paradoxes of developmental progress

A salient feature of the accounts of adolescents in this study was a sense of how pain had interrupted their *expected* developmental trajectory. At both an individual level and a broader societal level, an expected developmental trajectory was described as including continued yearly progression through school and the development of autonomy across adolescence. This overwhelming sense of interruption to adolescent life is exemplified below in Emily's description of how she was required to repeat a school year due to the difficulties associated with managing her chronic pain condition. She describes “missing the boat” and being placed on a different trajectory from her peers, which raises the question of whether or not she can ever return to her previous position in social and academic spheres.

When it changed dramatically, I went from doing school full time to not doing school for a whole year and then slowly re-introducing myself again and that caused a lot of issues itself because of friends and having to re-sit a whole year of school and everything, now that I'm back I'm in a different school year completely and all of my friends have just taken their GCSEs and so that was—it was hard—that makes you grow (Emily, 15 years).

As illustrated above, the displacement and disruption associated with repeating a school year extended beyond that of “just” academic progress, it disrupted the wider social context of Emily's life. Emily's previous shared experiences no longer matches those of her new peer group, and she had been isolated from the friends whom she had shared her school journey with across the years. She had not just been stopped in her tracks, she had been displaced onto an entirely different social and academic trajectory, one in which Emily had to learn to relate to new friends and adopt new understandings of what being a pupil in that year meant. This suggests that pain does not just interrupt the adolescents' trajectory or remove protective factors such as existing friendships, but that it fundamentally alters the adolescents' trajectory in terms of direction and destination. Emily's specific description of personal “growth” from this disruption indicates an ability to derive benefit from this interruption. Arguably, Emily's use of the term may actually suggest a sense of flourishing despite her pain condition. Such a change in trajectory is comprehensible when the enormity of the change that pain places on adolescent life is understood. Life is *“completely different, under a completely different subheading,” (James, 14 years)*.

Indeed, although adolescents talked about chronic pain interrupting their lives and expected trajectory, such impact was not wholly negative. As Emily explains below, reality is more complex and she describes how her friends were slowly “catching up” with her, suggesting a sense of enhanced emotional maturity. This suggests that although pain changes the adolescent trajectory, such alterations may actually be perceived as positive, depending on the metric of development applied.

I think I was trying to fight them at the beginning because I just wanted to be a normal teenager and muck about with my friends. But I think now it's better because now they've slowly going through that change and maturing and catching up with me so it's like I just got there first—now I'm a lot more mature than you but you're getting there soon so we'll go out again and be the same but I think it was just me getting there first and now they're starting to catch up (Emily, 15 years).

To some, Emily's perception of enhanced maturity may seem surprising as it contradicts extant findings in this area, which typically suggest a predominantly negative disruption to developmental progression in adolescents with chronic pain (Eccleston et al., 2008). And yet, Emily's experience of emotional enhancement was not unique in this study. Katie also discussed a sense of developmental superiority and advancement compared with peers in terms of her ability to manage emotions and deal with difficult situations. *“I'm better to be frank. I'm better than them which is a bit big headed but I have help and I have been through a lot more than they have been through,” (Katie, 12 years)*.

But what is it about the experience of pain in adolescence that places adolescents on this different and perhaps more accelerated, emotionally mature trajectory in certain domains of functioning? As Sophie describes below, perceptions of developmental enhancement reflect the unique and often intense experiences and situations associated with managing an often unexplainable and unanticipated chronic pain condition. This might include dealing with a multitude of health care professionals, receiving numerous, and sometimes conflicting diagnoses, in addition to undertaking a large number of invasive diagnostic tests.

I think in some ways I'm more mature cause I've had to go through quite a lot at quite a young age and I got different diagnosis and different tests and I think being ill makes you grow up faster and you grow up faster emotionally but not so much just in experiences (Sophie, 17 years).

Adolescents with chronic pain typically face a rapid and steep learning curve in terms of learning how to effectively communicate with health care professionals and importantly, how to advocate for their rights in situations that are characterized by inherent power differentials. A sense of growing up “emotionally” as Sophie describes above, extends to managing feelings around a disruption to the expected trajectory of adolescent life. Dealing with missed experiences translates into a more mature sense of how to deal with difficult emotions and perceived isolation from shared peer experiences. As highlighted by Ben below, not only are adolescents describing accelerated growth but also they are describing the acquisition and mastery of skills that they believe peers and even adults may not experience “*in a lifetime.*”

I think I've dealt with more things than my friends have dealt with in the past, since I've known them really because they've just had school and stuff whereas I've had to like deal with other things like not being able to get there, feeling upset, just feeling that I can't part take in things just not being able to walk, sometimes even being paralysed, so I think I've developed some skills that some people probably don't even use in a lifetime (Ben, 13 years).

These narratives highlight that describing adolescent development in terms of being simply ahead or behind in comparison with healthy peers is unsatisfactory. It oversimplifies the inherently idiopathic experiences of adolescents who experience pain in this study. As expressed by Sarah below, chronic pain does more than disrupt the trajectory, it offers an alternative way in which we can think about and “measure” development in adolescents with chronic pain. As Sarah highlights, she perceives a sense of difference from her peers because she is reliant on her mother for help when her peers are not.

Getting my independence, because I sort of needed my mum to help me do stuff whereas everyone else sort of ditched their mums, so it was harder to become more independent as I changed (Sarah, 16 years).

But, a reliance on parents in terms of “becoming independent” was not experienced universally by adolescents in this study. Despite the experience of pain and associated symptoms of impairment, Grace explains how her sense of autonomy has burgeoned in recent years, highlighting her sense of growing independence and critically, her ability to use it.

I've enjoyed going out more, because we live in a cool area and I'm allowed out more to town now and people don't treat me like a kid any more (Grace, 13 years).

Yet, the issue of granting of autonomy is more complex than alluded to above. In contrast with the existing ideas within the field of pediatric pain, Emily explains that it is not that her parents have withheld her autonomy, the issue is that she is unable to enact the autonomy that she has been granted. The issue is not the extent to which Emily feels independent in comparison with her peers, but whether Emily feels able to make use of the autonomy she has been provided.

I went from having all the independence and then not being able to use it cause I've been really ill but now I've been able to get it back and did good progress and mum and dad are not so worried about letting me go off on my own because before I would just collapse on the floor and my friends had to call them and say you'll have to come and pick up Emily cause she's really bad (Emily, 15 years).

To summarise, this second theme provides an understanding of how the experience of chronic pain can disrupt and alter adolescent developmental trajectories at an individual level. Importantly, the theme has highlighted that altered developmental trajectories for adolescents who experience chronic pain can include possibilities of both enhanced and delayed trajectories, suggesting more than a sense of delay.

## 4. Discussion

This idiographic study of adolescent development revealed that adolescents' development of identity, autonomy, and emotional maturity was invariably affected by the interruption associated with the experience of chronic pain. Expectations for adolescents' development were transformed, with trajectories being accelerated for some adolescents in particular domains and halted for other adolescents. Two themes emerged. One reflected the externally imposed lens placed on adolescents' identities through a clinical diagnosis and social perceptions, which was perceived to stunt, and sometimes even steal, their self-determination to identify as an adolescent rather than a diagnosis. Iatrogenic effects of clinical encounters, which often centre on disability rather than exploration of self beyond pain, were alluded to. And still, some adolescents described the importance they ascribed to holding onto a unique characteristic that defined them either despite their pain or because of it (eg, being a dancer). This provided a way for adolescents to identify as someone other than a “patient.” Key within this theme was the importance of describing adolescent development within a social context, reflecting the influence of relationships with others (eg, family, peers, and clinicians).

A second theme reflected challenges associated with drawing developmental comparisons between adolescents who experience chronic pain and those who don't. Adolescents described not simply an interruption in development but a fundamental change in its course and destination. Specifically, some described enhanced communicative skills and emotional development as a result of living with chronic pain. Pain equipped them with a unique sense of strength. Yet, other adolescents described their development as regressing or being suspended in time as a result of pain, denying them the ability to grasp and embrace autonomy. Consequently, adolescents' perceptions of their developmental progression were both invariably changed yet continually evolving as a result of their condition.

Regarding normative comparisons, our study findings are both consistent with and extend those of Eccleston et al.,^9^ which identified that 51% of adolescents with chronic pain perceived themselves to be developmentally behind same age peers in at least 4 areas of social development. Eccleston et al.^9^ identified 3 main domains of social development, independence, emotional adjustment, and identity formation, demonstrating that pain intensity negatively influenced all 3 domains. Although our findings corroborate how pain can challenge development in these core areas and development more broadly, the narrative approach elucidates the complexity and individual nature of the developmental progress of adolescents with chronic pain. In accordance with previous qualitative findings, our results confirm the difficulties experienced by youth with chronic pain in maintaining peer relationships and making developmental progress. However, our results reveal a more complex picture that is not limited to stunted development. Specifically, a developmental advancement in the ability to solve problems was observed in previous literature^9^ and was reflected in the experiences of some of the adolescents in this study. In contrast to extant research, our findings underscored the complexity of the disruption of chronic pain on adolescents' identity, identifying the possibility of *enhanced* personal growth and maturity in adolescents who experience chronic pain. Such findings are partially consistent with those of a recent study, which identified that high levels of benefit finding were reported by youth with chronic pain who reported the lowest levels of physical and psychological functioning.^36^ In the wider pediatric literature, studies have described the concept of post-traumatic growth in adolescents with cancer.^17^ Consistent with this literature, our study findings present a complex picture of accelerated and stunted development with resistance towards identity interruption and change. Participants were enhanced in terms of emotional maturity and navigating the health care system, but diminished in terms of age, smaller social networks, and independence (eg, Ben).

Study findings that concern an elevated developmental trajectory for some adolescents also warrant a brief discussion within the wider resilience literature. In particular, such findings provide an example of Masten's^22^ concept of “ordinary magic” that describes successful meeting of developmental milestones in youth despite adversity. For adolescents in this study, adversity refers to living with chronic pain and associated disability. Fitting with a recent review and theoretical conceptualisation of resilience in pain more broadly, our results support the importance of youth focusing on both recovery but also the ability to maintain positive well-being and functioning.^16^ These findings also highlight the broader social nature of development in the context of pain, focusing on the importance of how individuals perceive their development in relation to that of others (eg, peers) but also, how others perceive developmental progress and functioning of youth with chronic pain. The social nature of the developmental lens identified in this study resonates with a recently published ecological resilience-risk model for pediatric chronic pain,^7^ which specifically addresses individual and family factors regarding paediatric adaptation to chronic pain.

Throughout our study, adolescents situate their sense of self in relation to others, most often their peers. Fitting with existing research on peer relationships, a number of adolescents reported the onset of chronic pain to result in them having to “rethink friendships,”^12^ sometimes resulting in a loss of previously close friendships. Yet, this perceived sense of difference from peers was also experienced in a very different sense by other adolescents in our study. Consistent with a study of adolescents with type 1 diabetes, a number of adolescents in our study reported feeling more mature compared with peers.^6^ It could be that adolescents who reported enhanced emotional maturity may also have a more developed sense of empathy, regarding their ability to recognise and respond appropriately to emotions and suffering in others.^37^ It is also possible that because of the often extensive and typically negative psychological impact of pediatric chronic pain on adolescents and others, adolescents with chronic pain have greater experience dealing with difficult, emotionally charged situations than adolescents without chronic pain.

Although our results demonstrated that adolescents can have a surprisingly nuanced understanding of the complexity of their developmental status (eg, Sophie), it is important to be mindful that the accounts are narratives from adolescents whose “anchors” for understanding themselves and the world are constrained by their individualised repertoire of experiences. For example, Ben's account of, at the age of 13 years, mastering skills that “some people probably don't even use in a lifetime” is an assessment based on his narrative about himself and adulthood; it may not be shared by an external observer.

Moving beyond evidence of pain-specific influences on social development and identity in particular, our findings are in part resonant with those in the broader literature on paediatric long-term health conditions. Consistent with existing studies of youth with arthritis and type 1 diabetes, our findings highlighted that youth with pain described that their condition influenced their perception of the world and resulted in a sense of loss of identity for some.^6,23^ Yet, there are important differences in our findings compared with those of other studies, which may reflect the unique nature of chronic pain. In our study, this perceived loss of identity seemed to be due to social labelling as a “patient” or “disabled,” rather than the actual experience of pain or associated disability and resulting inability to undertake previously valued activities.^35^ For instance, in our study, Alice reported retaining her identity as a dancer despite the constraints placed on by living with chronic pain. Adolescents in our study also reported strength in the face of managing an often complex chronic pain condition, positioning themselves alongside youth with juvenile arthritis who reported a strong sense of self-identity. This is in contrast to youth with chronic fatigue syndrome who reported weak levels of self-identity.^15^

Current study findings are consistent with the assumption that the onset of a long-term condition can disrupt an individual's life course and perceptions of the world. Accordant with Frank's^14^ description of illness being able to disrupt an individual's life course through the loss of the map and the destination, adolescents in our study reported a sense of developmental disruption due to pain. It is important to note that Frank's work originates from the adult literature, and it is possible that this sense of disruption may differ for adults compared with adolescents for developmental reasons. Indeed, the adult chronic pain literature has clearly shown that chronic pain in adulthood does disrupt adult lives in numerous and typically negative ways.^1^ Over time, individuals work hard to negotiate new identities to manage this unwanted disruption to their lives. Yet for many adults (and women in particular), such changes in identity generation relate to their altered caring roles as parents and/or spouses,^3^ roles that adolescents who experience chronic pain rarely adopt during adolescence. Moreover, adolescents are typically experiencing the normative processes associated with identity development in addition to the specific challenges imposed on identity development due to living with chronic pain, highlighting a very different experience to that of adults with chronic pain.

Revisiting Frank's work, one particular aspect of disruption centred around identity development, a fundamental task of adolescence. Our findings illustrate how adolescent identity formation is a dynamic, multifaceted and long-lasting process,^11^ shaped through exploration of (dis)likes and various roles and grounded within their social–cultural context.^18,26^ Following Erikson's formulation of identity status on a continuum from diffusion to achievement, these study findings highlight that living with chronic pain does not necessarily preclude adolescents from reaching a desired stage in identity development. Although some adolescents appeared to experience an active struggle or crisis of compromise between societal expectations and their own capabilities, reflective of Erikson's moratorium status (eg, Emily), others demonstrated clear signs of identity achievement on their own terms (eg, Alice).

This study has notable strengths. It is the first study to adopt an idiographic approach to studying adolescents' perceptions and experiences of social development within the context of living with chronic pain, enabling an in-depth exploration of developmental issues. Second, we ensured that rigour was used in the analytical process through consideration of important quality criteria for assessing qualitative work such as transparency regarding the analytical process.^10,31^ Nonetheless, the study includes a number of limitations. First, the age range of study participants (12–17 years) was narrow. Although this age range reflects the adolescent period, recent discussion in the child development literature has considered the possibility of an extended adolescence.^30^ Consequently, it would be useful for future studies to recruit adolescents from a wider age range (eg, 11–25 years) to more fully explore individuals' perceptions of developmental progression. Second, it is important to acknowledge that the sample, all of whom had attended a tertiary-level residential pain program, reported high levels of disability and a long pain duration. As such, it is important to be cautious when considering the applicability of results to other pain populations.

Future research can helpfully adopt a broader social perspective concerning the study of adolescent development in the context of pain. For example, it would be useful to elicit the perspectives of parents, siblings, peers, teachers, and clinicians, in addition to the adolescent themselves. Second, longitudinal research is needed with a focus on eliciting accounts from individuals at different time points across the adolescent developmental period and treatment/recovery process. This would provide information concerning the dynamic changes in adolescents' perspectives and experiences regarding development in the context of chronic pain across this period and elucidate the potential impact of an individual's pain journey on identity development. Consistent with findings in juvenile arthritis,^23^ it is possible that the nature of identity formation in adolescents with chronic pain may differ between those whose pain began in adolescence vs those whose pain began in childhood.

This novel idiographic study focused on exploring how adolescents with chronic pain make sense of their own development highlighted the complexity of the perception and negotiation of adolescent identity. For some adolescents, identity development was a wholly personal phenomenon, whereas others described how their sense of identity development was viewed by others through a broader social external lens. Findings highlighted the disruption of chronic pain in terms of altering adolescents' developmental trajectories but also the complexity and individualised nature of this disruption. Overall, results provide a more nuanced perspective of thinking about developmental progress in the context of adolescent chronic pain and suggest some challenges with drawing normative comparisons with healthy peers.

## Disclosures

The authors have no conflict of interest to declare.

This work was funded by the Royal United Hospitals Bath NHS Foundation Trust. This research study was grant supported by charitable donated funds from the Royal United Hospitals Bath NHS Foundation Trust.
